# Colloidal lithography as a novel approach for the development of Ni-nanocavity insulin sensor

**DOI:** 10.1038/s41598-022-15283-7

**Published:** 2022-06-30

**Authors:** Ivana Šišoláková, Ondrej Petruš, Jana Shepa, Zdeněk Farka, Andrej Oriňak, Renáta Oriňaková

**Affiliations:** 1 Department of Physical Chemistry, University of P.J. Šafárik in Košice, Moyzesova 11, 040 01 Košice, Slovak Republic; 2 Institute of Materials Research, Slovak Academy of Sciences, Watsonova 47, 040 01 Košice, Slovak Republic; 3 Department of Biochemistry, Faculty of Science, Masaryk University, Kamenice 5, 625 00 Brno, Czech Republic

**Keywords:** Electrochemistry, Screening

## Abstract

In this study, a highly sensitive, fast, and selective enzyme-free electrochemical sensor based on the deposition of Ni cavities on conductive glass was proposed for insulin detection. Considering the growing prevalence of diabetes mellitus, an electrochemical sensor for the determination of insulin was proposed for the effective diagnosis of the disease. Colloidal lithography enabled deposition of nanostructured layer (substrate) with homogeneous distribution of Ni cavities on the electrode surface with a large active surface area. The morphology and structure of conductive indium tin oxide glass modified with Ni cavities (Ni-c-ITO) were characterized by scanning electron microscopy (SEM) and atomic force microscopy (AFM). The diameter of the resulting cavities was approximately 500 nm, while their depth was calculated at 190 ± 4 nm and 188 ± 18 nm using AFM and SEM, respectively. The insulin assay performance was evaluated by cyclic voltammetry. Ni-c-ITO exhibited excellent analytical characteristics, including high sensitivity (1.032 µA µmol^−1^ dm^3^), a low detection limit (156 µmol dm^−3^), and a wide dynamic range (500 nmol dm^−3^ to 10 µmol dm^−3^). Finally, the determination of insulin in buffer with interferents and in real blood serum samples revealed high specificity and demonstrated the practical potential of the method.

## Introduction

The pancreas is considered one of the most important glands in the human body^1^. It consists of endocrine and exocrine glands^2^ and is responsible for the production of an essential anabolic hormone, namely insulin^3,4^, which is released by the β-cells of the endocrine pancreas^5–7^. The main functions of insulin include the regulation of glucose metabolism^3^ as well as the initiation of glucose uptake by the cells^8^. A normal fasting insulin level in blood is 25 mIU L^−1^ (0.86 µmol dm^−3^)^5^. Insulin also affects the metabolism of fatty acids and amino acids^3^. Inadequate pancreatic function, which influences the production or action of insulin, leads to serious health effects, specifically the development of diabetes mellitus (DM)^9–11^.

DM is characterized by hyperglycaemia, i.e., high blood glucose level^12–14^ caused by insulin deficiency or its improper secretion^15^. Patients with diabetes may suffer from various cardiovascular system complications, kidney impartment, blindness, or limb amputations^10,12,16^. Three main types of diabetes are recognized at present. Type 1 diabetes is known as insulin-dependent diabetes, in which autoimmune destruction of β-cells leads to absolute insulin deficiency^17^. Noninsulin-dependent diabetes, i.e., type 2 diabetes^18^, results from insulin resistance or defective insulin production^19^. Insulin resistance in this type of diabetes is linked to an unhealthy lifestyle and overweight^8^. Another type of diabetes is gestational diabetes mellitus, which is associated with hyperglycaemia diagnosed during pregnancy^20,21^.

Since diabetes is one of the most common diseases worldwide^22^, the development of fast, inexpensive, efficient, and accurate sensors for early disease diagnosis and effective therapy is essential. Notably, electroanalytical sensors have been considered as suitable tools for the diagnosis of diabetes^23^. Currently, electrochemical sensors are widely used due to their fast, and accurate detection without the need for demanding instrumentation and trained staff. The most common applications of electrochemical sensors are detection of dopamine^24^, hydrogen peroxide^25^, heavy metals^26^, and nowadays are electrochemical sensors also more focused on the detection of viral diseases^27^ and bacteria^28^.

Electrochemical insulin determination takes just a few seconds. Moreover, electrochemical sensors are inexpensive, highly sensitive, and selective^29,30^. Our previous studies focused on the modification of screen-printed carbon electrodes by multi-walled carbon nanotubes (MWCNTs) and various metal nanoparticles (NPs), e.g., Cu, Co, Ni, and Zn^5,29,30^. Notably, MWCNTs increased the surface area of the working electrode. In addition, we investigated the catalytic activity of metal NPs toward insulin oxidation. It was found that Ni was suitable material for electrode modification due to its high catalytic activity toward insulin oxidation, which was attributed to the formation of NiOOH in the alkaline medium. However, the limitation of this sensor was its poor reproducibility due to the utilization of MWCNTs.

Hence, the objective of this work was to develop a highly reproducible, disposable Ni-cavities modified electrode with an active surface area comparable to that of a carbon electrode modified by a combination of NiONPs and MWCNTs^4^. We focused on the modification of conductive glass as the electrode substrate with Ni cavities prepared via colloidal lithography. The prepared nonocavities ensured the considerable expansion of the active surface area in comparison to bare unmodified conductive glass or conductive glass modified by an even Ni layer. The morphology of the electrodes was examined by scanning electron microscopy (SEM) with energy-dispersive X-ray (EDX) and atomic force microscopy (AFM). The active surface area of the modified electrodes was determined via cyclic voltammetry (CV) using the Randles–Ševčík equation. The surface area of the prepared Ni-cavities electrode was compared with those of unmodified and Ni layer-modified electrodes. CV was also employed to study the electrochemical properties of the prepared electrodes, when Ni cavities modified electrode exhibited high sensitivity (1.032 µA µmol^−1^ dm^3^), a low detection limit (156 µmol dm^−3^), and a wide dynamic range (500  to 10 µmol dm^−3^). All electrochemical measurements were conducted in a phosphate-buffered saline (PBS) solution simulating the presence of Cl^−^ ions at the same concentration as that in the body fluids. CV was also used to determine insulin in the presence of interferences as well as in the blood serum with no influence of interferences, demonstrating the potential of the sensor for the analysis of real samples.

## Experimental

### Chemicals and reagents

Xylene, acetone, ethanol, nickel sulfate heptahydrate, nickel chloride heptahydrate, boric acid, hydrogen peroxide, sulphuric acid, potassium chloride, potassium hexacyanoferrate trihydrate, nitric acid, sulfuric acid, d-(+)-glucose, l-ascorbic acid, uric acid, sucrose, sodium dodecyl sulfate, l-threonine, l-tyrosine, and dimethylformamide (DMF), and human blood serum were obtained from Sigma-Aldrich (Missouri, USA). Recombinant human insulin and PBS (D8662, sterile filtered) were purchased from MP Biomedicals (California, USA) and Biowest (Kansas City, USA), respectively. Sodium hydroxide was obtained from Milan Adamik (Bratislava, Slovakia). Polystyrene nanospheres (5% w/v in water) with a diameter of 518 ± 15 nm were acquired from microParticles (Berlin, Germany). Indium tin oxide (ITO) glass slides with less than 7 Ω·sq^−1^ resistance were purchased from Zhuhai Kaivo Optoelectronic Technology Co. (Zhuhai, China).

Ultrapure water (18.2 MΩ cm^−1^) was used for the preparation of all solutions and for cleaning the samples. Prior to each electrochemical measurement, the insulin solutions were freshly prepared by dissolving powdered insulin in a 0.1 mol dm^−3^ NaOH solution in PBS (pH 13). The solutions of sucrose, uric acid, ascorbic acid, glucose, tryptophan, and tyrosine were prepared in the same way. Blood serum samples were prepared by dissolving calculated powdered insulin to obtain required insulin concentrations in pure human blood serum without dilution. pH of obtained blood serum used for insulin determination was adjusted with 0.1 mol dm^−3^ NaOH and the calculated amount of powdered insulin was dissolved in it to obtain required insulin concentration in human blood serum.

### Instruments

All electrochemical measurements were performed using Metrohm AUTOLAB PGSTAT302N potentiostat (Utrecht, Netherlands) controlled using a software Nova 1.10, combined with a conventional three-electrode system consisting of platinum, Ag/AgCl (saturated KCl), and Ni nanocavity-modified ITO glass as the counter, reference, and working electrodes, respectively. All electrochemical measurements were conducted at room temperature and atmospheric pressure.

The structure and surface morphology of the electrodes were characterized by an SEM CrossBeam system (AURIGA Compact) with EDX analysis (ZEISS Germany) as well as by AFM (Dimension FastScan Bio with ScanAsyst-Air probe; Bruker, USA). The phase composition of the Ni-cITO was analyzed by XRD PhilipsX’ PertPro, CuKα radiation (Philips, Netherlands).

### Modification of ITO glass by Ni nanocavities

Monodispersed polystyrene spheres (PS) were used as received without further purification to prepare a monolayer colloidal mask via spin coating. The sample was prepared employing a previously described procedure^31^. Briefly, a microscope glass slide was carefully inserted into a 3:1 solution of H_2_SO_4_ and H_2_O_2_ and tempered at 80 °C for 1 h. Following the chemical treatment, the glass slide was very hydrophilic. This was critical for the formation of a highly precise, hexagonal closest packed monolayer. Subsequently, the glass slide was washed several times with ultrapure water, dried gently in a stream of nitrogen, and mounted to the spin coater holder. PS colloidal solution (80 μL) was dropped onto the microscopic glass slide (2.5 × 7.5 cm^2^) and subjected to spin coating at 1600 rpm with acceleration of 100 rpm s^−1^ for 5 min. The amount of used PS suspension covered whole surface of glass slide, what represent the area approximately 19 cm^2^. The PS-modified glass slide was then carefully immersed in a beaker containing deionized water, and a monolayer of the PS spheres was transferred to the air/water interface. A small amount (10 μL) of 5% sodium dodecyl sulfate (SDS) was added to the water caused gradient of surface tension along two liquids interface and mass transfer, in our case transfer the PS spheres close to each other. This effect is called Marangoni or Gibbs-Marangoni effect. This resulted in the formation of a close-packed colloidal mask with minimal structural defects. Finally, the colloidal mask was transferred onto the ITO glass slide by slowly picking it up from the water surface and drying at 50 °C.

The electrochemical deposition of the Ni nanocavity layer was conducted using a solution containing 0.6 mol dm^−3^ NiSO_4_, 0.1 mol dm^−3^ NiCl_2_ and 0.3 mol dm^−3^ H_3_BO_3_. An ITO/PS-modified glass slide was used as the working electrode, Pt foil with an area of ~ 1 cm^2^ was employed as the counter electrode, and a saturated calomel electrode was used as the reference electrode. Due to the imperfect colloidal mask and minimal differences in the glass dimensions, the electrochemical deposition was conducted under a constant potential at *E* =  − 1 V with a variable deposition time. The deposition was manually stopped after the *I–t* dependence reached the maximum. Following the deposition of the Ni films, the Ni-c-ITO glass slide was immersed in DMF for two days to dissolve the PS spheres completely. Finally, the samples were rinsed with acetone, ethanol, and deionized water, dried in a stream of nitrogen, and sintered at 250 °C for 30 min to increase the adhesion of the Ni films on the ITO glass slide. As the reference, the Ni film without any nanocavities was prepared by the same electrochemical and thermal procedures; however, the colloidal mask was not present on the ITO glass slide.

### Results and discussion

### Surface morphology characterization

#### Scanning electron microscopy analysis

**Figure 1:**
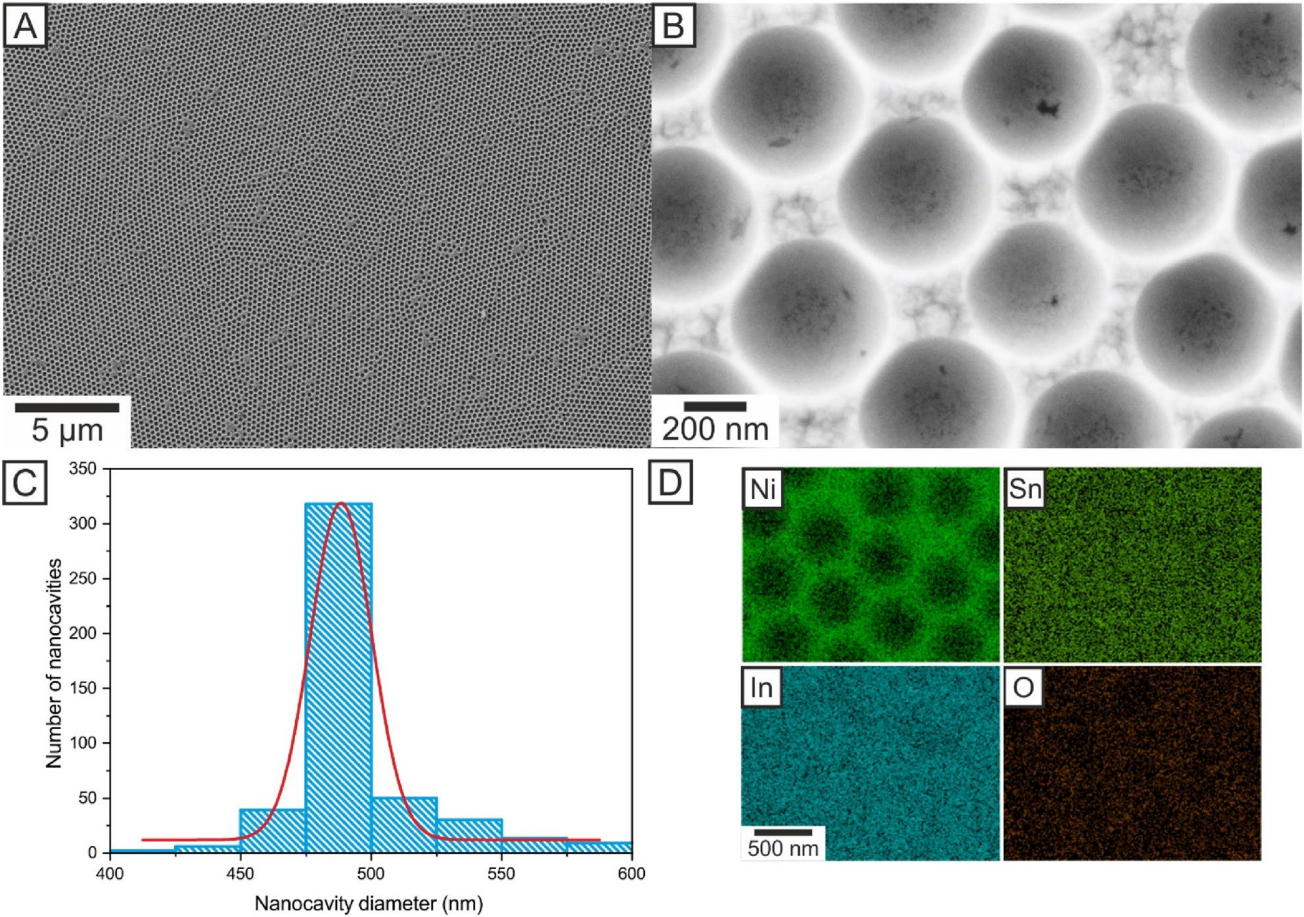
SEM images of the Ni nanocavity film at ×5000 magnification (**A**) ×150,000 magnification (**B**) with cavity size distribution (**C**) and EDX elemental distribution maps For Ni, Sn, In and O (**D**).

Nickel nanocavity arrays were prepared by electrochemical deposition of Ni through the voids of the close-packed monolayers of a colloidal mask consisting of PS spheres on ITO glass slides. The diameter of the resulting nanocavities approximately corresponded to the diameter of the templating spheres, whereas the depth of the voids was roughly set to the radius of the PS spheres. Figure 1A,B show the Ni nanocavities at different magnifications after the removal of PS in DMF. The resulting Ni nanocavity films formed a homogeneous hexagonal closest packed structure with minimal defects and vacancies over a large area (Fig. 1A). The average diameter of the nanocavities was calculated to be 498 ± 38 nm, which was consistent with the nanocavity size distribution obtained from the analysis of SEM images using the ImageJ software (Fig. 1C). However, the appearance of nanocavities with diameters in the range of 400–600 nm was observed in the distribution histogram. The depth of the nanocavity from SEM image can be calculated from following trigonometry equation (Eq. 1):

1 $$t={r}_{s}\pm \sqrt{{\left({r}_{s}\right)}^{2}{-\left({r}_{c}\right)}^{2}}$$

where *t* is the thickness of the layer, *r*_*s*_ is the radius of PS spheres and *r*_*c*_ is the top radius of cavity measured from SEM image. The choice of the sign ± depends on the whether the film is thicker or thinner than the radius of used PS spheres. For example, if the top radius is 500 nm, then we obtain the thickness of Ni nanocavity film 326 nm or 192 nm. According to the SEM image (Fig. 1B) where are clearly visible the sidewalls of the nanocavity, we have chosen in the calculation the sign minus. The depth of Ni nanocavity was calculated to 188 ± 18 nm. The deviations were attributed to the presence of larger or smaller spheres in the colloidal solution as well as to the type of method used for thresholding during SEM image analysis in ImageJ. The EDX maps of In, Ni, Sn and O are shown in Fig. 1D. As can be seen the EDX maps of Ni (73.2%) correspond to the SEM image (Fig. 1B). The map of Ni analysis copy outline of the cavity. Presence of Sn (2.5%) In (10.4%) and O (13.7%) on whole surface is due to conductive layer of Indium oxides on the glass slide.

#### Atomic force microscopy analysis

**Figure 2:**
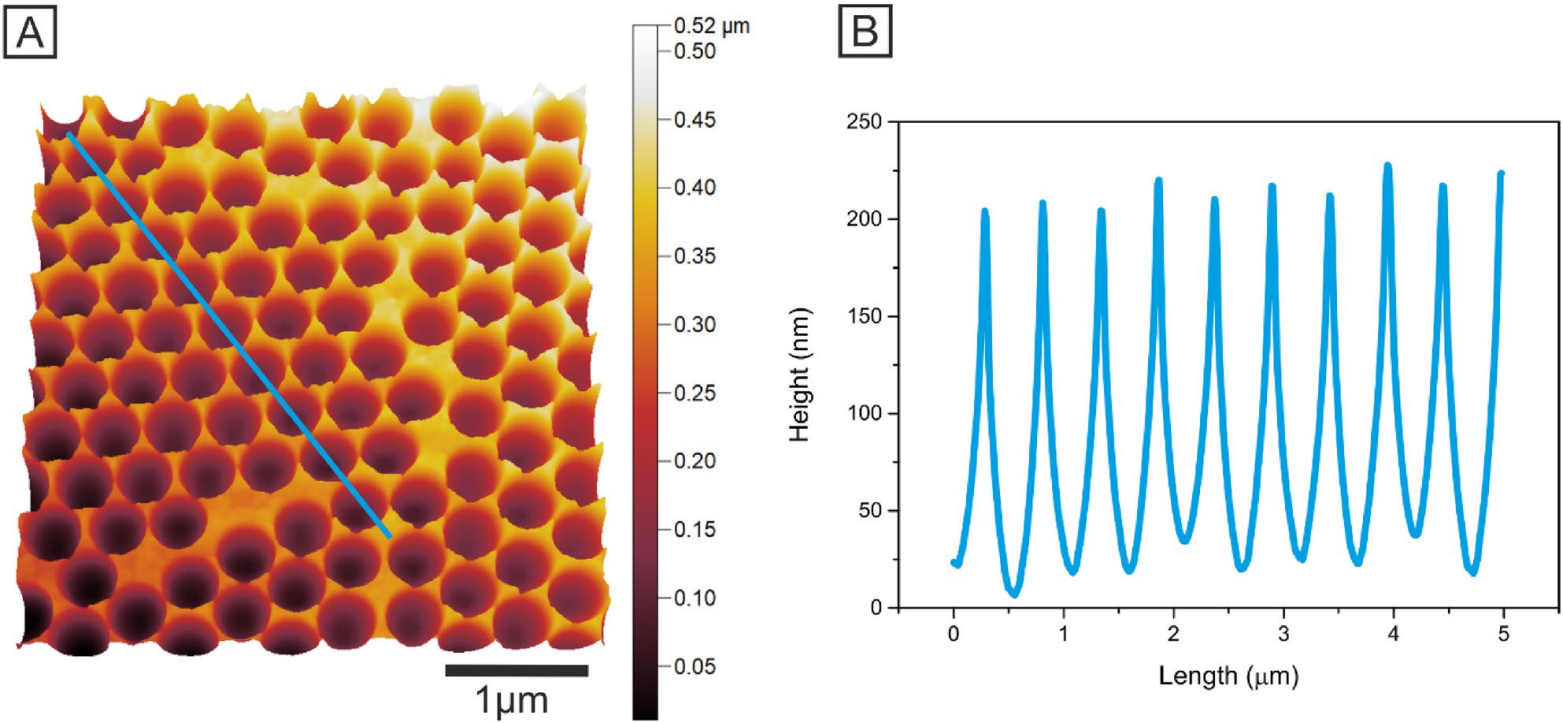
AFM image of Ni nanocavities (**A**) and the height profile obtained from the AFM image along the marked blue line (**B**).

The topographic tapping mode AFM image revealed the successful formation of highly ordered hexagonal closest packed Ni nanocavity arrays (Fig. 2A). The surface profile measured along the blue line is shown in Fig. 2B, demonstrating the depth of the Ni nanocavities of 190 ± 4 nm, which was in good agreement with the SEM analysis, where the calculated depth was 188 ± 10 nm. The minor differences between the AFM measurements and SEM image analysis were attributed to a tip convolution artefact and imperfect thresholding during SEM image evaluation. The measurement of the real surface area was also compared with the calculations based on the SEM images. The number of nanocavities with a geometric surface area of 25 µm^2^ was 102, whereas a real surface area was 45.45 µm^2^. This corresponded to an increase in the surface area by approximately 77%. At the same geometric area, the real surface area calculated using the obtained SEM/EDX images was 31.90 µm^2^. Such a significant difference in the measurement was attributed to the utilization of optimal conditions in the calculations, where only one structural deformation was included and that the bottom of the nanocavity is not covered by Ni.

#### XRD structural analysis

**Figure 3:**
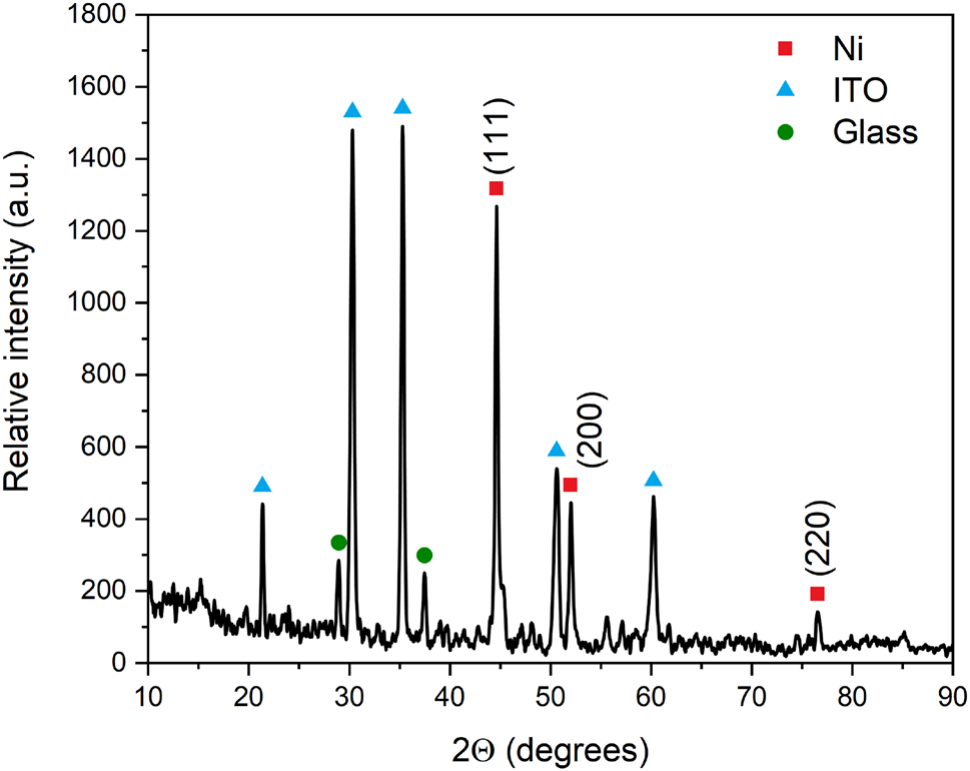
XRD spectrum of Ni nanocavity deposited on ITO glass slide.

The crystallinity of the Ni-c-ITO electrode was observed by XRD patterns in Fig. 3. The presence of Ni was indicated by 3 sharp peaks at 2Θ = 44.63°, 52.05° and 76.55° characteristic for Ni(111), Ni(200) and Ni(220), respectively^32^. The peaks at the 2Θ = 21.37°, 30.32°, 35.30°, 50.61° and 60.24° corresponds to the ITO(211), ITO(222), ITO(400), ITO(440) and (622) planes respectively^33,34^. The small peaks observed at the 2Θ = 28.95° and 37.45° corresponds to the components of glass substrate. In the XRD analysis was no presence of NiO peaks because Ni is passivated only on the surface and the amount of NiO is too low for XRD analysis^35^.

#### Active surface area determination

**Figure 4:**
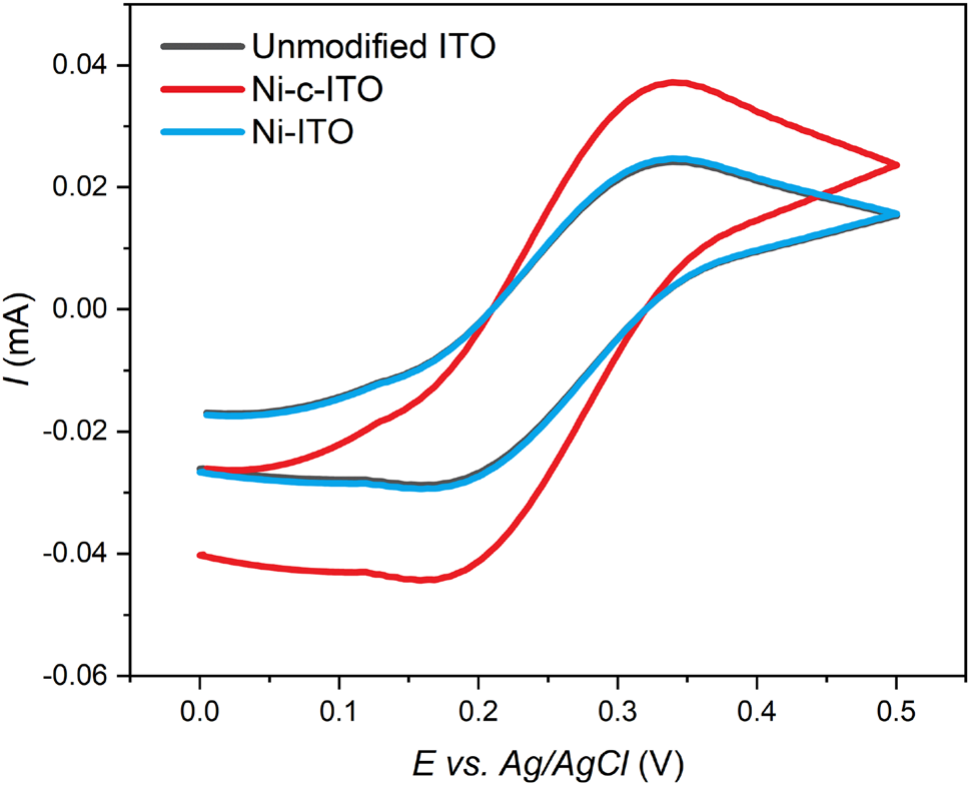
Cyclic voltammograms for unmodified ITO glass—black curve, Ni-ITO—blue curve, and Ni-c-ITO—red curve in 5 mM K_3_[Fe(CN)_6_]/K_4_[Fe(CN)_6_] in 1 M KCl at 50 mV s^−1^.

The active surface area of Ni cavities-modified ITO glass (Ni-c-ITO) was determined via CV using 5 × 10^–3^ mol dm^−3^ K_3_[Fe(CN)_6_]/K_4_[Fe(CN)_6_] in 1 mol dm^−3^ KCl (Fig. 4). The Randles–Ševčík equation (Eq. 2) was used for the calculation:

2 $${I}_{p}=0.4463\cdot nFAC{\left(\frac{nFvD}{RT}\right)}^{1/2}$$

where *I*_*p*_ is the current maximum in A, *n* refers to the number of transferred electrons, *A* indicates the electrode area in cm^2^, *C* is the concentration of the electroactive species in mol cm^−3^, *v* denotes the scan rate in V s^−1^, *D* is the diffusion coefficient in cm^2^ s^−1^, *R* is the gas constant in J K mol^−1^, *F* is the Faraday constant in C mol^−1^, and *T* is the temperature in K.

The calculated active surface area of Ni-c-ITO was compared with those of unmodified and Ni layer-modified (Ni-ITO) ITO glass samples. The current response of unmodified ITO glass and Ni-ITO was nearly identical. The rapid increase in the current response was observed in the case of Ni-c-ITO. In this case, the electroactive surface area increased almost twofold. The electroactive surface areas of unmodified ITO, Ni-ITO, and Ni-c-ITO were 1.87, 1.95, and 2.89 cm^2^, respectively. Thus, Ni-c-ITO exhibited the highest active surface area and provided the highest number of active sites at the electrode surface, which led to an efficient insulin determination.

### Electrochemical characterisation

#### Electrochemical behaviour of insulin at unmodified ITO, Ni-ITO, and Ni-c-ITO

**Figure 5:**
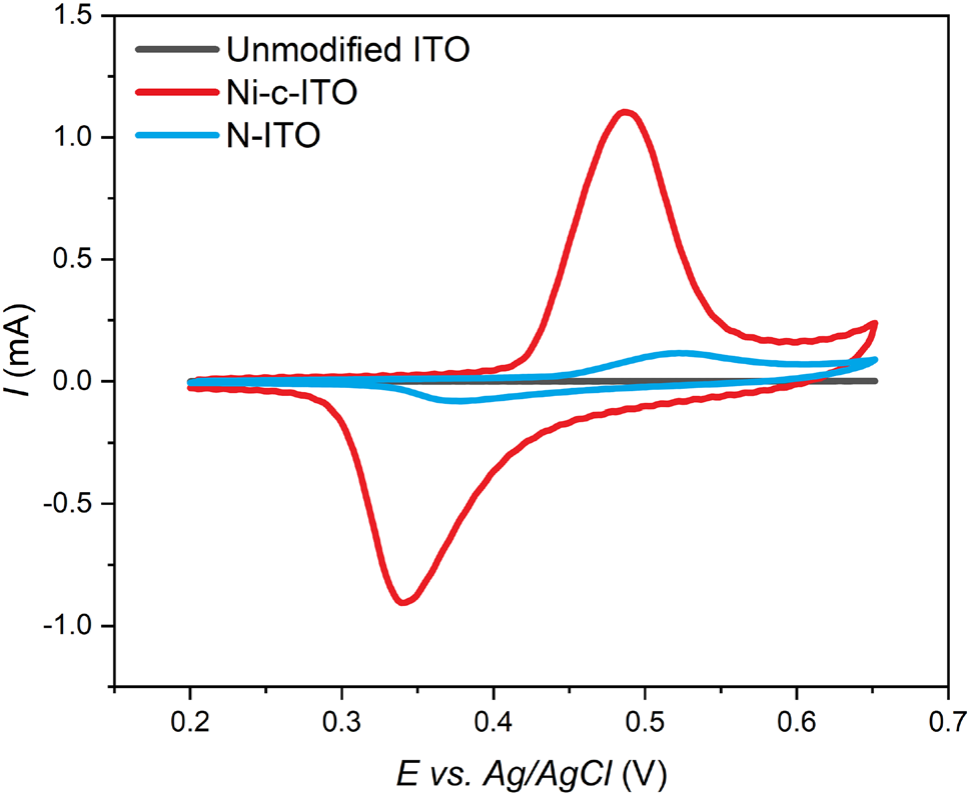
Cyclic voltammograms of unmodified ITO glass (black curve), Ni-ITO (blue curve), and Ni-c-ITO (red curve) in the presence of 5 × 10^–6^ mol dm^−3^ insulin in 0.1 mol dm^−3^ NaOH and PBS at 50 mV s^−1^.

To study the electrochemical behaviour of unmodified ITO, Ni-ITO, and Ni-c-ITO, we have registered the CV curves of the electrodes in the presence of 5 × 10^–6^ mol dm^−3^ insulin in 0.1 mol dm^−3^ NaOH and PBS at 50 mV s^−1^ (Fig. 5). As can be seen (Fig. 5, black line), no oxidation or reduction peaks were detected for unmodified ITO. Following the deposition of the Ni layer at the ITO surface, a current response corresponding to insulin oxidation was observed with the potential of *E* = 0.52 V (Fig. 5, blue line). This confirmed the catalytic activity of Ni towards insulin oxidation. The highest current response was noted in the case of Ni-c-ITO. This outcome was attributed to the significant increase in the active surface area of the electrode, which led to the establishment of a higher number of active sites on its surface. The current peak potential corresponding to insulin oxidation on Ni-c-ITO shifted to a more negative value of *E* = 0.47 V, which further verified the electrocatalytic activity of Ni towards insulin oxidation. The potential decrease was advantageous due to the oxidation of biological elements in the blood at higher potential values (*E* =  ~ 0.8 V)^36^. The difference between the anodic and cathodic peak potentials was 130 mV, indicating the irreversibility of the system. Based on these results, Ni-c-ITO was used for further analysis.

#### Study of the kinetics of insulin oxidation at Ni-c-ITO

**Figure 6:**
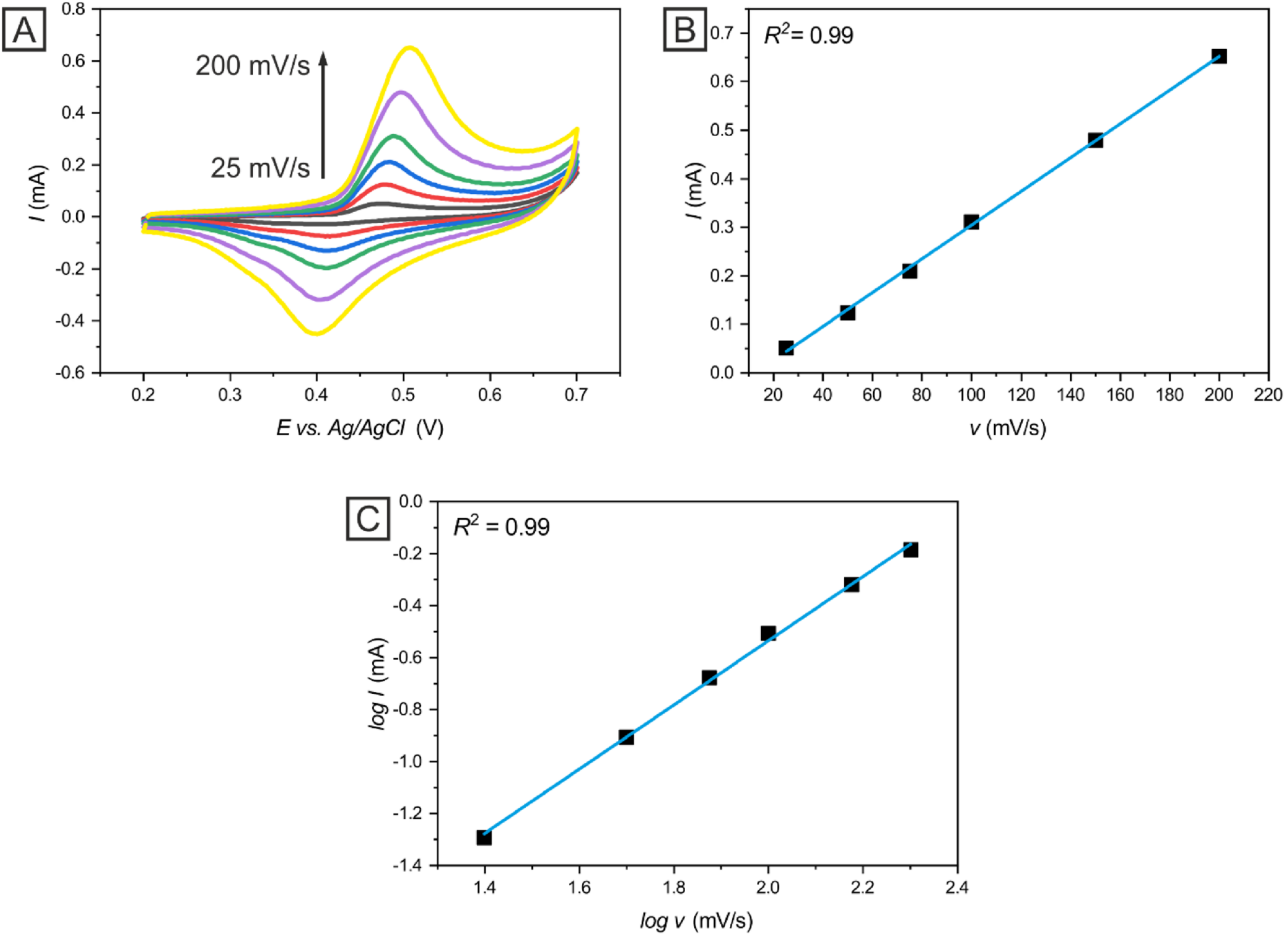
Cyclic voltammograms of 2 × 10^–6^ mol dm^−3^ insulin on Ni-c-ITO at different scan rates (**A** (25 mV s^−^ to 200 mV s^−1^)). The dependency of peak current on the scan rate (**B**) and the dependence of peak current of log I on log v (**C**).

In an effort to elucidate the insulin oxidation mechanism, we conducted CV measurements of 2 × 10^–6^ mol dm^−3^ insulin at different scan rates (25–200 mV s^−1^). Figure 6A shows the cyclic voltammograms of 2 × 10^–6^ mol dm^−3^ insulin in 0.1 mol dm^−3^ NaOH and PBS on Ni-c-ITO at various scan rates with linear regression (Fig. 6B,C). Figure 6 also demonstrates the dependence of the maximum current value corresponding to the insulin oxidation (*I*) on the scan rate (*v*) in Ni-c-ITO (Fig. 5B) as well as the dependence of log *I* on log *v* in Ni-c-ITO (Fig. 6C). Both dependences were fitted with a linear function. In both cases, the scan rate and the square root of the scan rate were linear with the *R*^2^ = 0.99. The linear dependency of log *I* on log *v* exhibited the following linear regression equation: log *I* = 1.23 log *v* − 3.04, which indicated an adsorption process based on the adsorption of the insulin onto the working electrode. Biomolecule adsorption is usually conditioned by providing enough hydroxyl groups on the surface. Therefore, the alkali solution displays a catalytic influence on the electrochemical detection of various biomolecules. Since Ni-c-ITO was sintered at 250 °C for 30 min, the thin layer of NiO was presumably created during the sintering process^37^. As shown in a previous study^38^, the mechanism of insulin oxidation on NiO-modified electrodes is initiated by the oxidation of NiO to NiOOH (Eq. 3). Subsequently, direct insulin oxidation and NiOOH reduction results in the generation of oxidized insulin and Ni(OH)_2_, respectively (Eq. 4)^38^. The electrocatalytic particles NiOOH are generated in alkaline pH which strongly catalyses the insulin oxidation on NiO modified electrodes.

3 $$NiO+{OH}^{-}\leftrightarrow NiOOH+{e}^{-}$$

4 $$NiOOH+insulin \leftrightarrow Ni{(OH)}_{2}+product$$

#### Study of the electroanalytical performance characteristics

**Figure 7:**
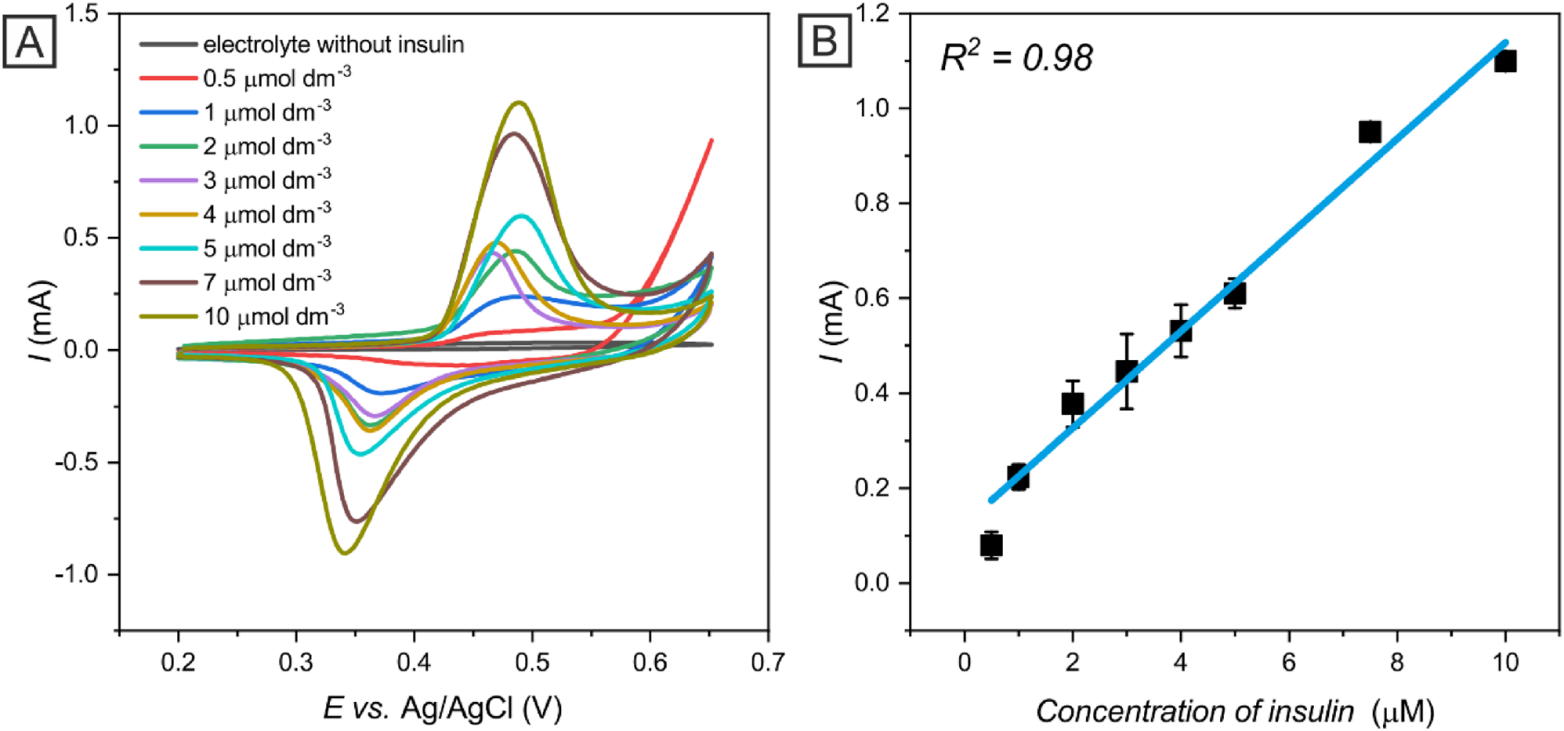
Cyclic voltammograms obtained for electrolyte without insulin and various insulin concentrations in 0.1 mol dm^−3^ NaOH and PBS on NiO-c-ITO at a scan rate of 50 mV s^−1^ (**A**). The dependency of the peak current on the insulin concentration fitted by a linear function (**B**).

Electroanalytical performance characteristics of Ni-c-ITO were studied by CV in PBS containing 0.1 mol dm^−3^ NaOH and a specified amount of insulin at a scan rate of 50 mV s^−1^. The peak current increased linearly with the concentration of insulin (*R*^2^ = 0.98) (Fig. 7A). The results were fitted by a linear function to assess various analytical performance characteristics of Ni-c-ITO, including the limit of detection (LOD), sensitivity, and linear range (Fig. 7B). The LOD was calculated from linear regression using the following equation (Eq. 5)^39^:

5 $$LOD=\frac{3{S}_{a}}{b}$$

where *S*_*a*_ indicates the standard deviation of the response and *b* is the slope of the calibration curve. Ni-c-ITO displayed a wide linear range from 0.5 to 10 µmol dm^−3^, with a low LOD of 0.156 µmol dm^−3^ and high sensitivity of 1.032 µA µmol^−1^ dm^3^. The electroanalytical performance characteristics of the Ni-c-ITO electrode were compared with the performance characteristics of other previously reported electrodes modified by metal NPs for insulin determination (Table 1). Notably, compared to other methods, the electrochemical sensor described herein enabled faster and cheaper analysis. Ni-c-ITO exhibited comparable sensing properties and linear concentration range of NP-modified electrodes.

**Table 1:** Comparison of analytical parameters of nano-modified electrodes used for insulin determination.

Electrode	Linear range (µmol dm^−3^)	LOD (µmol dm^−3^)	Sensitivity (µA µmol^−1^ dm^3^)	Method	References
PSiMPs	1–5	0.371	–	Spectrophoto-metry	^40^
Carbon quantum dots/GCE	0.005–0.010	0.001	0.80	EIS	^41^
GO/AuNPs/ TX-100	0.002–0.3	0.001	0.64	Colorimetry	^42^
Chitosan-CNTs/GCE	0.1–3	0.30	135	Amperometry	^43^
MWCNTs/planar carbon electrode	0.25–1.6	0.25	–	Cyclic voltammetry	^44^
CoOxNPs/GCE	0.0001–0.015	0.00001	0.084	Amperometry	^45^
Ni(OH)_2_NPs/Nafion-MWCNTs	Up to 10	0.085	5	Amperometry	^46^
Ni-c-ITO	0.5–10	0.156	1.032	Cyclic voltammetry	This work

### Analysis of the stability, selectivity, and reproducibility of Ni-c-ITO

**Figure 8:**
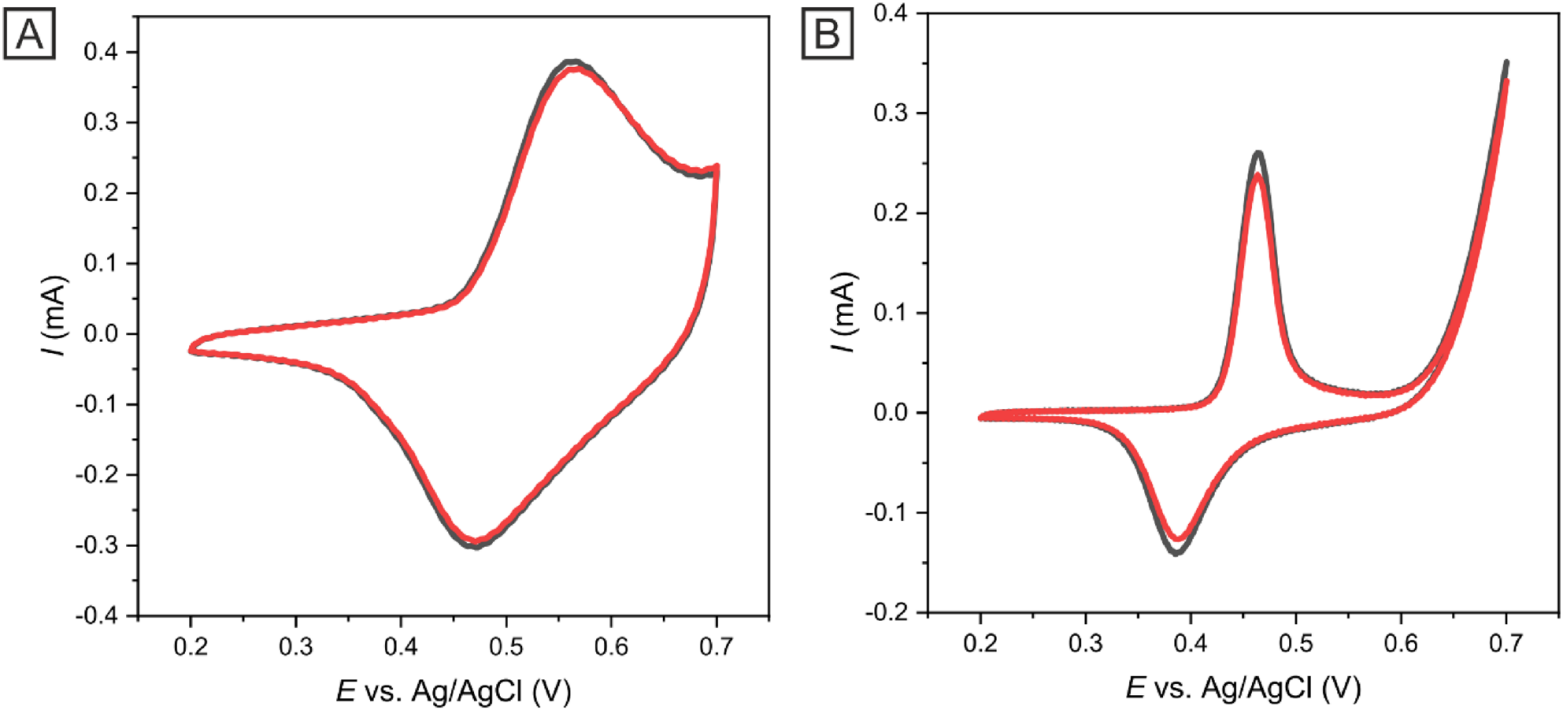
CV measurements used for the evaluation of the stability of Ni-c-ITO using 1 × 10^–3^ mol dm^−3^ K_3_[Fe(CN)_6_]/K_4_[Fe(CN)_6_] in 1 mol dm^−3^ KCl at 50 mV s^−1^ (**A**) and 2 × 10^–6^ mol dm^−3^insulin in 0.1 mol dm^−3^ NaOH and PBS (**B**). Black line (1st measurement) and red line (50th measurement).

Stability studies focus on the capability for minimal drifting of CV peak current values with measurements in aqueous media over time. To determine the Ni-c-ITO stability, repeated CV cycles in model analyte (1 × 10^–3^ mol dm^−3^ K_3_[Fe(CN)_6_]/K_4_[Fe(CN)_6_] in 1 mol dm^−3^ KCl) and insulin solution in 0.1 mol dm^−3^ NaOH and PBS in potential range of 0.2–0.7 V at 50 mV s^−1^ were conducted; up to 50 CV cycles were performed as shown in Fig. 8. After 50 cycles, a decrease in the current response by less than 4% in model analyte (Fig. 8A) and 6% in insulin solution was observed. These results demonstrated that the Ni-c-ITO sensor was remarkably stable even after a large number of measurements (50 cycles). Thus, Ni-c-ITO could be utilized for the development of inexpensive and effective sensors.

**Figure 9:**
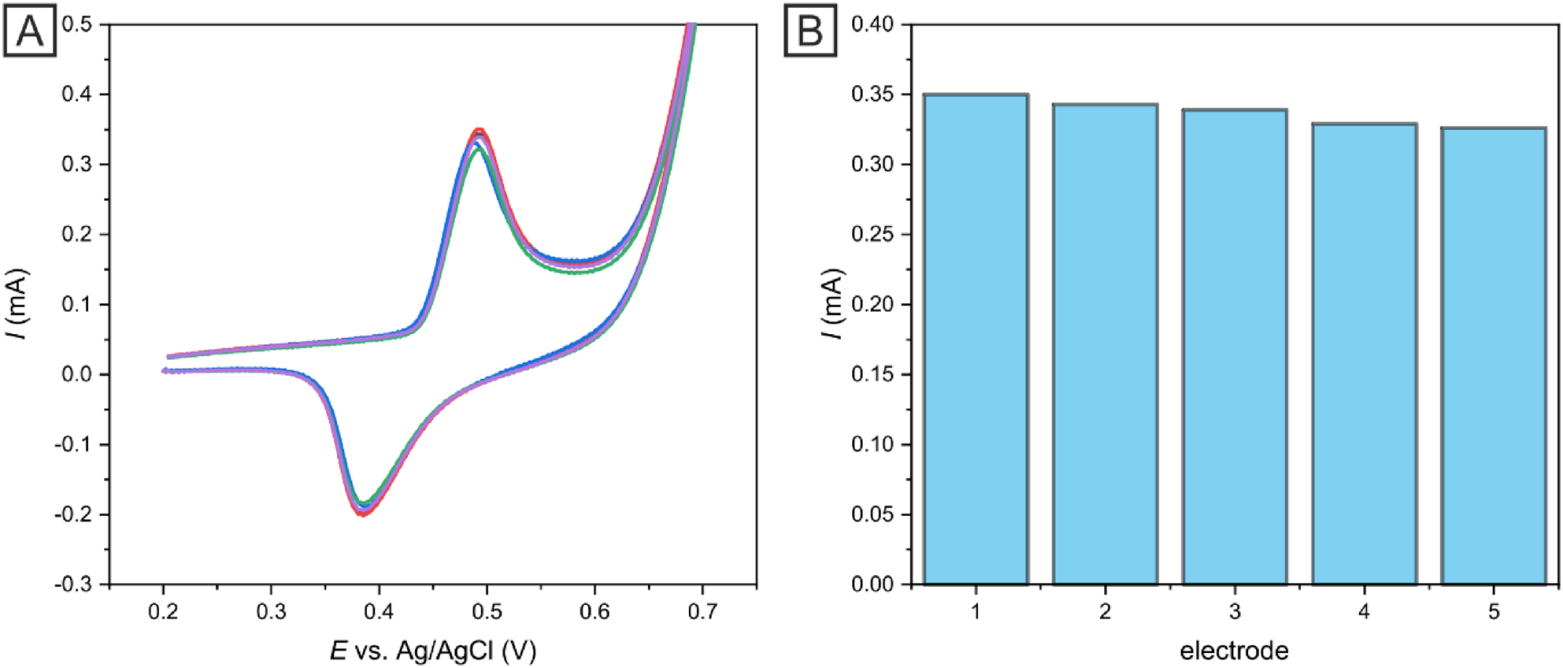
CV measurements used for the evaluation of reproducibility of Ni-c-ITO electrodes using 2 × 10^–6^ mol dm^−3^ in 0.1 mol dm^−3^ NaOH and PBS at 50 mV s^−1^ (**A**). The fabrication reproducibility of Ni-c-ITO in the different toward 2 × 10^–6^ mol dm^−3^ in 0.1 mol dm^−3^ NaOH and PBS at 50 mV s^−1^ (**B**).

To investigate the reproducibility of the Ni-c-ITO fabricated in the same way, the measurements of the amperometric response are further performed for five selected electrodes toward insulin (2 × 10^–6^ mol dm^−3^). As illustrated in Fig. 9, the amperometric responses toward glucose oxidation at different electrodes are almost identical with a relative standard deviation (RSD) of only 0.88%, attributing to the high reproducibility of the Ni-c-ITO sensors.

**Figure 10:**
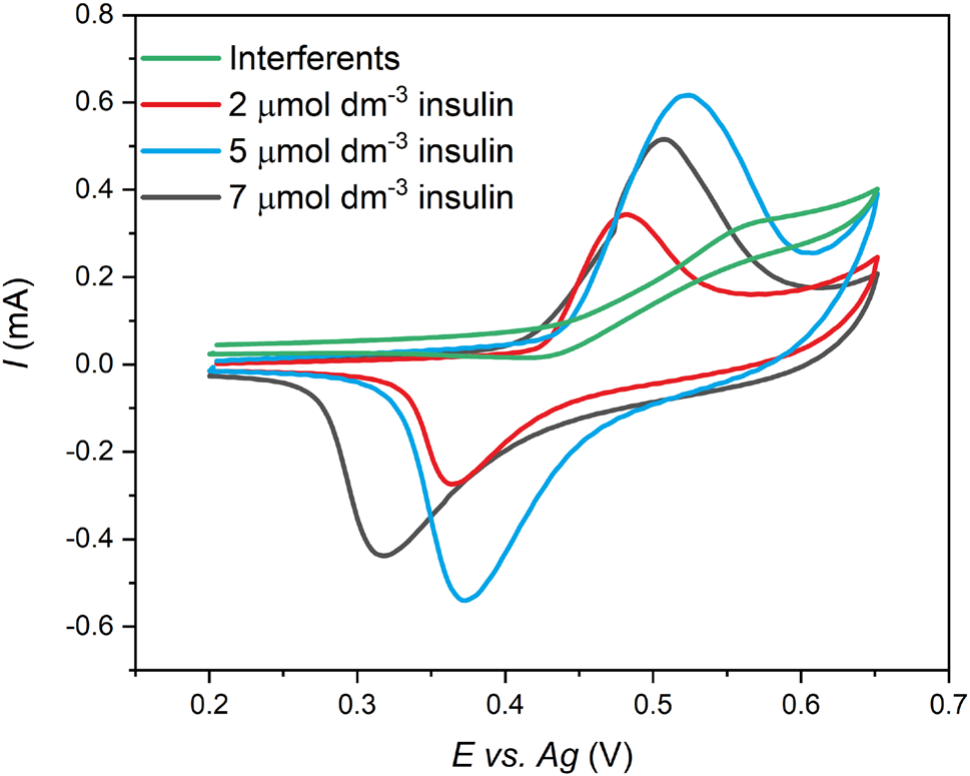
CV curves obtained in the presence of different interferences in the 0.1 mol dm^−3^ NaOH and PBS (5 × 10^–3^ mol dm^−3^ glucose, 0.1 × 10^–3^ mol dm^−3^ ascorbic acid, 0.1 × 10^–3^ mol dm^−3^ sucrose, 83 × 10^–6^ mol dm^−3^ threonine, 78 × 10^–6^ mol dm^−3^ tyrosine and 0.5 × 10^–3^ mol dm^−3^ uric acid) without insulin (green line) and with 2 × 10^–6^ mol dm^−3^ (red line), 5 × 10^–6^ mol dm^−3^ (black line), and 7 × 10^–6^ mol dm^−3^ (blue line) insulin at 50 mV s^−1^.

The selectivity of Ni-c-ITO was studied by CV using an electrolyte in the presence of interferences (5 × 10^–3^ mol dm^−3^ glucose, 0.1 × 10^–3^ mol dm^−3^ ascorbic acid, 0.1 × 10^–3^ mol dm^−3^ sucrose, 83 × 10^–6^ mol dm^−3^ threonine, 78 × 10^–6^ mol dm^−3^ tyrosine and 0.5 × 10^–3^ mol dm^−3^ uric acid). The selectivity was also assessed in the blood serum. As mentioned previously, all electrochemical measurements in this work were performed in PBS, which exhibited the same concentration of Cl^−^ ions as in the human blood. The green line in Fig. 10 corresponds to the CV curve of the solution containing the above-mentioned interferences in the absence of insulin. As can be seen in Fig. 10, no oxidation peak was observed at the potential corresponding to insulin oxidation on Ni-c-ITO. Figure 10 also shows the CV curves of solutions containing various interferences and 2 × 10^–6^, 5 × 10^–6^, and 7 × 10^–6^ mol dm^−3^ insulin. The current peak linearly increased with the insulin concentration also in the presence of interferences.

**Figure 11:**
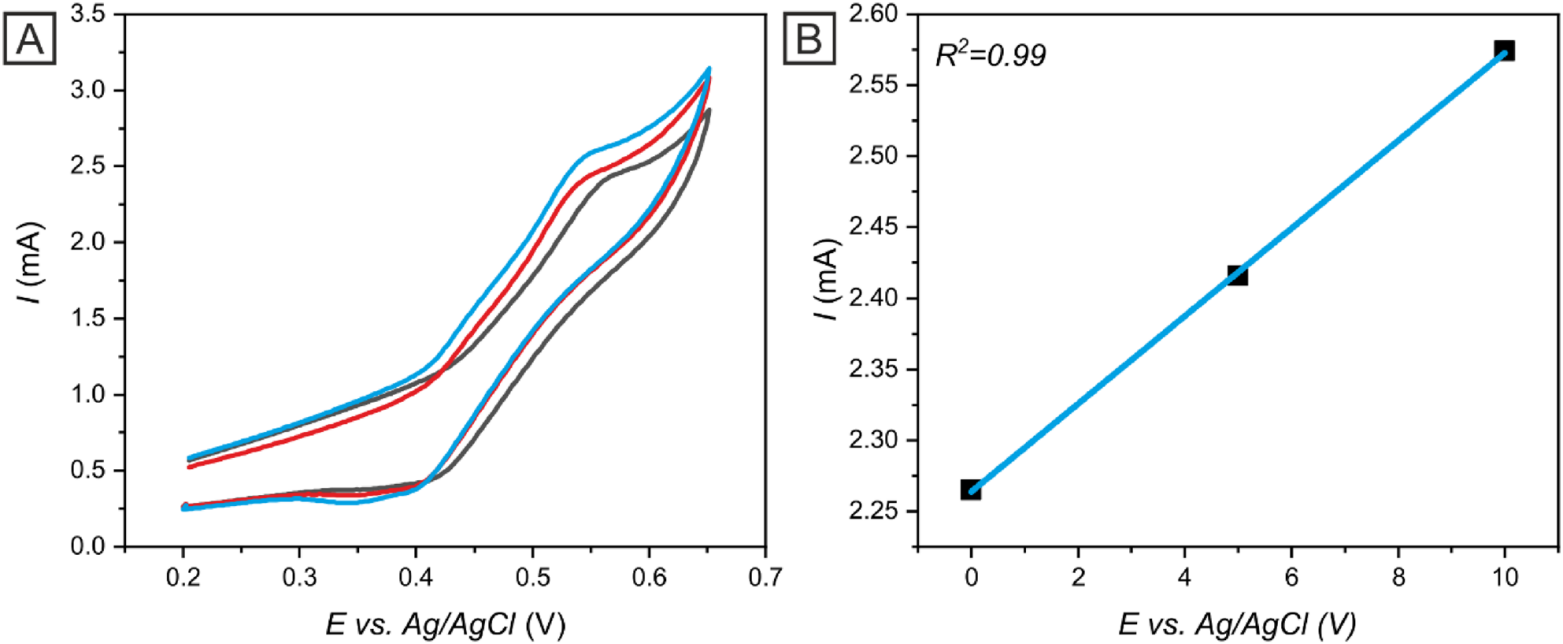
Cyclic voltammograms of pure blood serum (black line) and blood serum samples containing 2 × 10^–6^ mol dm^−3^ (red line) and 5 × 10^–6^ mol dm^−3^ (blue line) insulin on Ni-c-ITO at 50 mV s^−1^ (**A**). The dependency of the peak current on the insulin concentration fitted by a linear function (**B**).

To assess the applicability of Ni-c-ITO in real samples, the electrodes were utilized for the determination of insulin in human blood serum, which acted as the electrolyte in CV measurements. We examined the current response of the pure blood serum sample as well as of samples containing 2 × 10^–6^ and 5 × 10^–6^ mol dm^−3^ insulin (Fig. 11). The insulin oxidation peak was observed at the potential of *E* = 0.48 V and linearly (*R*^*2*^ = 0.99) increased with the rising insulin concentration in the blood serum. Therefore, electroanalytical performance characteristics were calculated for prepared electrode also in real samples. The LOD was calculated (Eq. 4) as 0.25 µmol dm^−3^ and sensitivity was determined as 0.31 µA µmol^−1^ dm^3^. Based on these results, Ni-c-ITO was established as a promising candidate for the detection of insulin in real samples because the low LOD includes also the normal fasting insulin level in human blood (0.86 µmol dm^−3^)^5^.

## Conclusions

Ni-c-ITO prepared via colloidal lithography was applied as an electrochemical sensor for insulin detection. The morphology of Ni-c-ITO was investigated by SEM with EDX analysis as well as by AFM. Compared with bare ITO and ITO modified with a Ni layer, the active surface area of Ni-c-ITO increased twofold, which resulted in an enhancement of the electrocatalytic activity. The prepared electrode exhibited favourable analytical characteristics, including a low LOD (0.156 µmol dm^−3^), high sensitivity (1.032 µA µmol^−1^ dm^−3^), and reproducibility (RSD = 0.88%) which were determined by CV measurements. Thus, Ni-c-ITO as a novel electrochemical sensor with homogeneous and reproducible surface Ni distribution as well as large active surface area was developed. Importantly, Ni-c-ITO acted as a selective sensor for the detection of insulin in blood serum, enabling its future applications in clinical diagnosis.

## Data Availability

The datasets used and/or analysed during the current study are available from the corresponding author on reasonable request.
